# Eclampsia—Poisoning by Opium

**Published:** 1866

**Authors:** M. W. Townsend


ART. III—*Eclampsia—Poisoning by Opium.* By M. W. Townsend.

At 9 P. M., November 30th, 1865, I saw Mrs. M. S., aged 20, in her first pregnancy, at the sixth month. Dr. J. O. Loomis, a stranger to me, was in attendance. The patient was a very anæmic lady, who had had for the preceding few days œdema of the extremities, face and eyelids, a scanty renal secretion, and a distressing headache. I learned that at about 5 P. M. she had been convulsed, and that three or four convulsions occurred previous to my visit, for which she had been treated with saline enema. The urine, drawn by catheter, yielded under tests an extraordinary amount of albumen. No signs of uterine action.

Induction of labor and the exhibition of chloroform were advised on the grounds that the case was one of uræmic poisoning, dependent on passive congestion of the kidneys, which was the result of the pressure of a gravid uterus; that although there might be great hope for fœtus and mother, in a case of hysterical or epileptical convulsions, or in convulsions of reflex origin, there could be none for a fœtus of six months in uræmic poisoning, and little for the mother, unless promptly delivered; in which event, according to M. Blot, Herr Braun and others, we might expect the kidney to recover its elimination of urea—that chloroform meantime would control the convulsions and nullify the reflex disturbance attending delivery.

The attendant physician objected to the advice, and proposed to "leave her to the 'vis medicatrix naturæ' of Cullen!" Other advice was sought of Drs. Horace Clark and J. W. Craig, and under the advice of the former the membranes were punctured and a large amount of amniotic liquor was discharged. One hour after, patient being under the influence of chloroform, no evidence of uterine action. At midnight, Dr. Craig arriving, another examination was made, revealing evidence of labor-pains; os uteri patulous, and readily admitting the index and easily yielding to a second finger; it was determined that an effort at delivery should be made. Fortunately the left foot of the fœtus was within the grasp of the two fingers used as forcep blades, and was readily brought down, followed by the right foot and pelvis. The patient was delivered without violence or undue effort, of both child and placenta. Chloroform was used occasionally during this procedure, and afterward, immediately upon indications of an impending paroxysm, and kept up only so far as to cause the premonitory symptoms of the paroxysm to disappear.

After delivery an enema was given and the bowels moved freely; a small quantity of stimulus was given and ordered to be repeated according to the discretion of the attending physician. Acetate of potassa grs. xx, in spts. nire. ʒj, once in four hours, and fluid food a teaspoonful once in two hours, completed the prescriptions.

During the day—December 1st—patient recovered consciousness, swallowed readily, talked of her sickness, and when not disturbed slept quietly. In the evening Dr. Craig used catheter and left patient sleeping. No appearance of coma.

December 2d, 7 A. M. Dr. Craig saw patient; remarked more restlessness, and left a small anodyne powder which was administered. At 10 A. M. I saw her, and there being considerable restlessness gave the attending physician three powders of one-sixth grain each of morphia, and advised him to administer one every four hours, as long as indications for use of morphia lasted, and no longer. At this visit the patient's appearance was favorable, as evidenced by the regular respiration, the good character of the circulation, and the voiding of eight or ten ounces of urine by catheter, which, when tested, yielded a much diminished quantity of albumen according to volume.

At 10 P. M. I found Mrs. S. comatose, would open the eyes when called, and when roused thoroughly answered a few questions and recognized her friends; pupils were contracted to the last possible degree; pulse small, not very much diminished in frequency; respirations infrequent, and when allowed to rest a moment would almost cease, but when shaken or spoken to in a loud tone more frequent for three or four moments; skin moist, cold and pale; catheter used and a full pint of urine voided. In a few minutes voluntary respiration ceased. Resort was had to Marshal Hall's method, and by it respiration was sustained for five hours. At times she respired voluntarily, and a marked improvement was noticed a half hour after the alternate, prone and supine positions of the body were commenced.

The character of the coma, the fact that she could be roused to consciousness when I first saw her, the condition of her pupils, the fact that she was kept alive for five hours by artificial respiration, her condition at the exhibition of the last powder, show the case to be one of poisoning by morphia.

That the uræmic intoxication made her more susceptible to the power of opium might not be denied, but that it was not the cause of death is evidenced by the free secretion of urine, her recovery from uræmic coma more than twenty-four hours before, the absence of the convulsions, the sudden invasion of the coma, and the almost entire absence of the general indications of uræmia. To attribute to chloroform any share of the result would be gratuitous, as the article had not been used for about thirty hours.

It is proper in this connection to say, that an emetic course at 10 o'clock P. M. was entirely out of question, impossible; there was scarcely any excito-motor power when I first saw her at my last visit. The morphia had been given rather more than two hours before, in solution.

I wish to mention also, that during the past year I saw a case of poisoning by opium—a half ounce of laudanum having been given per anum, which was saved by Hall's method. Respiration had ceased, the pulse was barely perceptible, but a resort to artificial respiration commenced seven hours after the enema, and after all else had been tried resulted in recovery from the effects of the poison, although it was six hours before the patient could be trusted to voluntary breathing.

---

At the Students' Medical Society at Edinburgh much discussion has occurred of the results of acupressure. It is stated that Prof. Syme will try the method of temporary ligature, described by Mr. Churchill in a recent number of the *London Lancet.*

